# Three Novel *Clostridia* Isolates Produce *n*-Caproate and *iso*-Butyrate from Lactate: Comparative Genomics of Chain-Elongating Bacteria

**DOI:** 10.3390/microorganisms8121970

**Published:** 2020-12-11

**Authors:** Bin Liu, Denny Popp, Nicolai Müller, Heike Sträuber, Hauke Harms, Sabine Kleinsteuber

**Affiliations:** 1 Department of Environmental Microbiology, Helmholtz Centre for Environmental Research—UFZ, 04318 Leipzig, Germany; liu.bin@ufz.de (B.L.); denny.popp@ufz.de (D.P.); heike.straeuber@ufz.de (H.S.); hauke.harms@ufz.de (H.H.); 2 Department of Biology, University of Konstanz, 78457 Konstanz, Germany; nicolai.mueller@uni-konstanz.de

**Keywords:** novel clostridial species, carboxylate platform, medium-chain carboxylates, branched-chain carboxylates, anaerobic fermentation, reverse β-oxidation

## Abstract

The platform chemicals *n*-caproate and *iso*-butyrate can be produced by anaerobic fermentation from agro-industrial residues in a process known as microbial chain elongation. Few lactate-consuming chain-elongating species have been isolated and knowledge on their shared genetic features is still limited. Recently we isolated three novel clostridial strains (BL-3, BL-4, and BL-6) that convert lactate to *n*-caproate and *iso*-butyrate. Here, we analyzed the genetic background of lactate-based chain elongation in these isolates and other chain-elongating species by comparative genomics. The three strains produced *n*-caproate, *n*-butyrate, *iso*-butyrate, and acetate from lactate, with the highest proportions of *n*-caproate (18%) for BL-6 and of *iso*-butyrate (23%) for BL-4 in batch cultivation at pH 5.5. They show high genomic heterogeneity and a relatively small core-genome size. The genomes contain highly conserved genes involved in lactate oxidation, reverse β-oxidation, hydrogen formation and either of two types of energy conservation systems (Rnf and Ech). Including genomes of another eleven experimentally validated chain-elongating strains, we found that the chain elongation-specific core-genome encodes the pathways for reverse β-oxidation, hydrogen formation and energy conservation, while displaying substantial genome heterogeneity. Metabolic features of these isolates are important for biotechnological applications in *n*-caproate and *iso*-butyrate production.

## 1. Introduction

Speciality chemicals such as *n*-caproate and *iso*-butyrate are valuable products of the carboxylate platform, with a broad range of potential applications in agriculture and industry [1,2,3]. For example, *n*-caproate can be used as promoter of plant growth and feed additive, or as precursor for the production of biofuels, lubricants, and fragrances [1,4,5,6,7]. Currently, *n*-caproate is mainly produced from vegetable oils such as palm kernel oil [8], though it can be produced from more sustainable feedstocks such as agro-industrial waste by anaerobic fermentation and microbial chain elongation [9,10]. Compared to linear carboxylates, branched-chain carboxylates such as *iso*-butyrate are of special interest for alternative applications due to their different physical properties, including higher viscosity, higher oxidative stability, and a lower boiling point [11]. For example, *iso*-butyrate can be used for the synthesis of texanol, which is a widely used coalescent for latex paints [2]. Currently, *iso*-butyrate is manufactured by acid-catalyzed Koch carbonylation of propylene, which is derived from fossil feedstock [2]. Microbial production of *iso*-butyrate from organic wastes or biomass residues is a more sustainable alternative as demonstrated by recent studies [12,13].

The metabolic process to produce *n*-caproate by anaerobic fermentation is called microbial chain elongation, also known as reverse β-oxidation. Some strictly anaerobic bacteria are known as chain elongators that use ethanol as electron donor providing reducing equivalents and acetyl-CoA for the elongation of acyl-CoA units, thereby increasing the chain length of carboxylates by two carbons with each cycle [1]. For example, *Clostridium kluyveri* has been well described to elongate short-chain carboxylates (e.g., acetate) to *n*-caproate through reverse β-oxidation with ethanol and acetate as sole carbon and energy sources [14]. Additionally, odd-numbered electron acceptors such as propionate can be elongated, leading to the formation of *n*-valerate and *n*-heptanoate [1,9]. The review paper of Angenent et al. highlighted the importance of the ethanol-based chain elongation pathway in biotechnology studies [1]. Additionally, chain elongation with lactate is getting increasing attention because some feedstocks (e.g., ensiled plant biomass) are rich in lactate, which is an important intermediate in the anaerobic breakdown of carbohydrates. To date, only few chain-elongating bacteria have been isolated that utilize lactate to produce *n*-caproate, including strains of *Megasphaera elsdenii*, *Megasphaera hexanoica*, *Pseudoramibacter alactolyticus*, and *Ruminococcaceae* bacterium CPB6. It has been assumed that the mechanism of chain elongation with lactate is similar to that described for chain elongation with ethanol [10,15]. However, insufficient knowledge has been generated yet on the physiology of lactate-based chain elongation from pure culture studies, and there is a lack of genome-level information to explore the genetic characteristics shared by chain-elongating bacteria. Previous studies have shown that *iso*-butyrate can be produced in methanol-based chain elongation [3,12,13]. The results suggested that *Clostridium luticellarii* might be responsible for the *iso*-butyrate formation during mixed culture fermentation, which was further tested by pure culture study of *C. luticellarii*, showing its ability to convert acetate and methanol to *iso*-butyrate [16]. However, the physiological reason for *iso*-butyrate formation in a chain elongation process has not been fully elucidated, particularly when lactate is the electron donor.

Recently, we reported on a complex bioreactor community that produced *n*-caproate from lactate-rich corn silage [17], and later a mixed culture producing *n*-caproate was enriched with lactate and xylan in a daily-fed bioreactor [18]. To investigate functional key species involved in *n*-caproate formation, we isolated several strains that are capable of converting lactate to *n*-caproate and *iso*-butyrate. For three isolates that turned out to represent novel species according to their 16S rRNA gene sequences, we performed whole genome sequencing and assembled the genomes with a short- and long-read sequencing hybrid approach as recently announced [19]. Further insight into the genomic and metabolic features of these strains may facilitate detailed understanding of lactate-based chain elongation.

The objectives of this study were to investigate the product spectrum of the three new lactate-consuming strains and to give insights into their metabolism based on their genomes. Batch experiments were conducted to explore the fermentation profiles with lactate. Functional genome annotation and phylogenomic analysis aimed at elucidating the genetic background of *n*-caproate and *iso*-butyrate production and the genetic heterogeneity between the three strains. To analyze the genomic diversity of the entire repertoire of chain-elongating species and to identify the core genes of chain elongation-related pathways and their conservation, we performed a comparative genome analysis by including eleven more genomes of experimentally validated chain-elongating species.

## 2. Materials and Methods

### 2.1. Enrichment, Isolation, and Identification of Lactate-Consuming Strains

Anaerobic fermentation broth from a caproate-producing reactor (38 °C, pH 5.5, and hydraulic retention time of 4 d) fed with corn silage was initially taken as the inoculum. Serum bottles (120 mL) with 45 mL mineral medium [18] containing 5 g/L lactic acid (initial pH 5.5) were inoculated with 5 mL of the sieved reactor broth (mesh size 2 mm). After replacing the headspace by N_2_/CO_2_ (80:20 in volume ratio, 100 kPa), the bottles were statically incubated at 37 °C in the dark. Liquid samples were collected every two weeks at the beginning, and later lactic acid was replenished when it had been consumed. Four successive transfers (1:10 dilution in fresh medium) were done, spanning more than 700 days.

A single bottle of the fourth transfer was used to isolate lactate-consuming strains. The culture was plated on complex agar (medium DSM104c with additional 5 g/L lactic acid) and incubated in an anaerobic chamber at 37 °C for two weeks. Colonies were picked and re-streaked three times for purification, and then transferred to liquid mineral medium bottles to determine their product spectrum. Further, the isolates that produced *iso*-butyrate and *n*-caproate were identified by Sanger sequencing of the 16S rRNA gene (details in Supplementary Methods). Based on 16S rRNA gene identity with their closest relatives, potential new species including the isolates designated as strains BL-3, BL-4, and BL-6 were selected for whole genome sequencing.

### 2.2. Batch Cultivation

To analyze the fermentation products from lactate at pH 5.5, batch cultures of isolates BL-3, BL-4, and BL-6 were run in mineral medium with lactate as sole carbon source and 0.05% yeast extract as described above. The bottles were inoculated with 5 mL seed cultures (optical density at 600 nm [OD_600_]~2), which were routinely cultivated in a complex medium (DSM 104c with extra 5 g/L of lactic acid added). The pH was adjusted to 5.5 with 1 M NaOH or 1 M H_2_SO_4_ after adding 50 mM lactic acid (85%, FCC grade; Sigma Aldrich, St. Louis, MO, USA) to the bottles. The cultivation bottles were statically incubated at 37 °C. Liquid samples were collected twice per week. After one week, lactic acid (75 mM) was added again to each bottle, and the pH was adjusted to 5.5 accordingly. All batch tests were carried out in duplicate.

For further investigation of the growth of isolate BL-4 on other carbon sources, anoxic bicarbonate-buffered freshwater medium pH 7.3 reduced with cysteine was used. The basal medium consisted of NaCl (1 g/L), MgCl_2_ (0.4 g/L), KH_2_PO_4_ (0.2 g/L), NH_4_Cl (0.25 g/L), KCl (0.5 g/L), CaCl_2_ (0.15 g/L), and Na_2_SO_4_ × 10 H_2_O (0.16 g/L) and was autoclaved for at least 30 min at 121 °C and 1 bar overpressure in a Widdel-flask. After cooling to room temperature under a stream of N_2_/CO_2_ (80:20), a separately autoclaved solution of NaHCO_3_ was added to a final concentration of 30 mM. Then each 1 mL of trace element solution SL13, 7-Vitamin solution and selenite-tungstate solution were added per liter medium (modified after [20,21,22,23]). Finally, the medium was amended with 0.4 mg/L resazurin as a redox indicator and filter-sterilized cysteine-HCl (3 mM final concentration) as reducing agent. In case the redox indicator of the medium did not turn colorless within 30 min of stirring under N_2_/CO_2_, 25 µM to 50 µM titanium(III)-nitrilotriacetic acid was added from a filter-sterilized stock solution to aid in establishing reduced conditions. This was the case for all pH 7.3-media used in this study. After the medium turned colorless, the pH of the medium was adjusted to pH 7.3 and the medium was thereafter dispensed into the cultivation vessels under N_2_/CO_2_. Where indicated, 0.05% yeast extract was added as an additional source of vitamins and amino acids. Strain BL-4 was cultivated in 25-mL tubes closed with rubber stoppers and filled with 10 mL medium at 37 °C. The OD_600_ was monitored over time with a Camspec tube photometer as described before [24].

### 2.3. Analytical Techniques

Liquid samples from the batch cultures at pH 5.5 were centrifuged for 10 min at 20,817× *g* (Centrifuge 5417R; Eppendorf, Hamburg, Germany) to remove cells. Acetate, lactate, propionate, *iso*-butyrate, *n*-butyrate, *n*-valerate, *n*-caproate, *n*-caprylate, and ethanol concentrations of the supernatant were determined in triplicate by high performance liquid chromatography (HPLC; Shimadzu Corporation, Kyoto, Japan) equipped with a refractive index detector RID-10A and a HiPlex H column together with a pre-column (Agilent Technologies, Palo Alto, CA, USA) as previously described [25]. HPLC samples from batch cultures at pH 7.3 were first acidified with 20 µL of 1 M H_2_SO_4_ prior to centrifugation, and the supernatant was analyzed by refractive index detection after separation on a Rezex RHM monosaccharide column with 30 mM sulfuric acid at 40 °C as described [24].

### 2.4. Gene Prediction and Annotation

We sequenced the genomes of the three isolates with the Oxford Nanopore Technologies MinION and the Illumina NextSeq platforms, and three complete genomes were constructed using a hybrid assembly approach as described previously [19]. Prediction and functional annotation of coding sequences (CDSs) was accomplished by the MicroScope automatic annotation pipeline [26]. Automatic annotations of selected CDSs were manually curated by comparing the protein sequences with the PkGDB, Swiss-Prot, TrEMBL, COG (Clusters of Orthologous Groups), EGGNOG (Evolutionary Genealogy of Genes: Non-supervised Orthologous Groups), FIGfams, and InterPro databases [26,27,28,29,30,31] by using the following methods: MaGe/Curated annotation, Syntonome RefSeq, Similarities SwissProt, Similarities TrEMBL, UniFIRE SAAS, UniFIRE UniRules, PRIAM EC number, FigFam, InterProScan and PsortB. COGNiTOR [32] was used to classify the CDSs into COG functional categories. CDSs classification into EGGNOG (v4.5.1) was performed by eggNOG-mapper v1.0.3 [29]. All these databases and tools are integrated in the MicroScope platform as described by Vallenet et al. [26]. Genomes of *Clostridium jeddahense* JCD, *Ruminococcaceae* bacterium CPB6, *Clostridium merdae* Marseille-P2935, *Megasphaera elsdenii* 14-14, *Eubacterium pyruvativorans* i6, *Megasphaera hexanoica* MH, *Caproiciproducens* sp. NJN-50, *Caproiciproducens galactitolivorans* BS-1, *Eubacterium limosum* KIST612, *Candidatus* Weimeria bifida, *Candidatus* Pseudoramibacter fermentans, and *Pseudoramibacter alactolyticus* ATCC 23263 were submitted to the MicroScope platform. The genome annotation of these strains available in the MicroScope PkGDB database was done by following the same procedures.

### 2.5. Phylogenetic Analysis and Taxonomic Classification

Phylogenetic analysis of 16S rRNA gene sequences was performed on the Phylogeny.fr platform [33]. According to the Nucleotide BLAST (Basic Local Alignment Search Tool) comparison result against the rRNA/ITS databases (16S ribosomal RNA sequences (Bacteria and Archaea)) of NCBI (National Center for Biotechnology Information) [34], the ten hits with the highest BLAST score for each isolate were selected. The 16S rRNA gene sequences of all selected strains were aligned using MUSCLE v3.8.31 with default settings [35]. After alignment, Gblocks v0.91b was used to remove ambiguous regions (i.e., containing gaps and/or poorly aligned) as described by Castresana [36]. The phylogenetic tree was reconstructed using the maximum likelihood method contained in PhyML v3.1 [37,38]. Robustness of tree topology was assessed by 100 bootstrap replicates. Finally, the tree was visualized by using TreeDyn v198.3 [39]. Besides the taxonomic classification of the genomes in MicroScope, GTDB-Tk v1.0.2 was used for taxonomic assignment to GTDB (Genome Taxonomy Database) [40] and the corresponding NCBI taxonomy.

A phylogenomic tree of strains BL-3, BL-4, BL-6, and other chain-elongating bacteria was calculated based on genomic similarity. The genomic similarity was estimated using Mash [41], which computes the distance between two genomes. This distance D is correlated to the average nucleotide identity (ANI) like: D ≈ 1-ANI. A neighbor-joining tree with clustering annotations was constructed. This clustering was calculated from all-pairs distances ≤0.06 (≈94% ANI) corresponding to the ANI standard to define a species group. The Louvain method for community detection was used for computing the clustering [42]. The ANI (OrthoANIu value) comparison of the genomes of the isolates to related genomes was calculated by an ANI calculator that improved the original OrthoANI (Average Nucleotide Identity by Orthology) algorithm by applying USEARCH instead of BLAST as described by Yoon et al. [43].

Default settings were used for all tools unless otherwise specified.

### 2.6. Pan-Genome Analysis

The interface Comparative Genomics of the MicroScope platform was employed to analyze the pan-genome, core-genome, and variable genome for our newly sequenced genomes and for all the available genomes of chain-elongating bacteria in the comparison. The MicroScope homologous gene families (MICFAM, protein sequence pairs with at least 80% amino-acid identity and 80% alignment coverage) [44] were considered for these analyses.

### 2.7. Data Availability

All data generated or analyzed during this study are included in this published article and its additional files. The full-length 16S rRNA gene sequences of the three isolates have been deposited in the European Nucleotide Archive (ENA, https://www.ebi.ac.uk/ena/browser/home) under BioProject PRJEB39379, with the accession numbers LR861112, LR861113 and LR861114. The genome data of the three isolates have been deposited in ENA under BioProject PRJEB36835, with Whole Genome Sequencing or Chromosome accession numbers CADDXC010000000, LR778134, and LR778135.

## 3. Results and Discussion

### 3.1. Isolation and Identification of Lactate-Consuming Strains

After incubation and four transfers of fermentation broth from a corn silage reactor with lactate as substrate, we enriched a mixed culture that produced acetate, *n*-butyrate, *iso*-butyrate, and *n*-caproate (Figure S1). Isolation of lactate-consuming strains was achieved by plating the mixed culture on complex agar to isolate single colonies. Eleven pure cultures were obtained as confirmed by 16S rRNA gene sequencing. In liquid culture using mineral medium, three strains (designated as BL-3, BL-4 and BL-6) were found to convert lactate to *iso*-butyrate and *n*-caproate. The 16S rRNA gene sequence of BL-3 was 96.8% identical to that of *Clostridium luticellarii* FW431, BL-4 was 93.8% identical to that of *Ruminococcaceae* bacterium CPB6, and BL-6 was 96.3% identical to that of *Clostridium jeddahense* JCD. According to the current species threshold (98.7%) based on 16S rRNA gene identity [45], these three strains can be assumed to represent novel species and were consequently selected for whole genome sequencing.

### 3.2. Conversion of Lactate to n-Caproate and iso-Butyrate in Batch Cultivation

**Figure 1:**
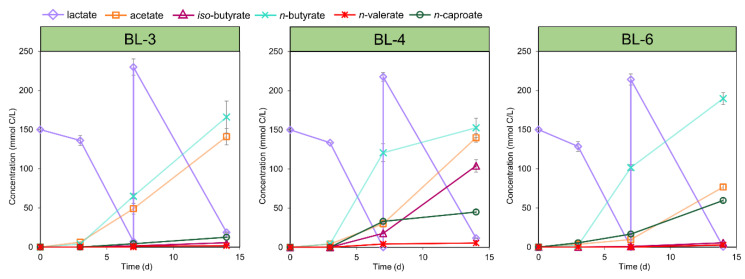
Fermentation products of strains BL-3, BL-4, and BL-6 during growth on lactate at an initial pH 5.5 and in the presence of 0.05% yeast extract. After one week, 75 mM lactic acid was replenished. Mean values of six measurements of duplicate batch cultures are given; error bars represent the standard deviation.

The pure culture batch experiments showed that all three newly isolated strains can convert lactate into acetate, *n*-butyrate, *iso*-butyrate, *n*-valerate, and *n*-caproate (Figure 1). Started at an initial pH 5.5, the three strains displayed different product spectra even though growing in the same mineral medium with lactate as the sole carbon source. Specifically, all three strains produced a large share of acetate (23% to 43%) and *n*-butyrate (34% to 57%), whereas propionate and *n*-caprylate were not detected. Based on the final concentrations (mmol C/L), strain BL-6 produced the highest proportion of *n*-caproate (18% for BL-6, 10% for BL-4, and 4% for BL-3) and strain BL-4 produced the highest proportion of *iso*-butyrate (23% for BL-4, 2% for BL-3, and 2% for BL-6), while only 1% *n*-valerate was produced by all three strains. As shown in Figure 1, the *n*-butyrate production rate decreased in cultures of BL-4 and BL-6 after the second spiking with lactate but was constant in the culture of BL-3. Simultaneously, the *iso*-butyrate production rate increased in BL-4 and the *n*-caproate production rate increased in BL-6. This indicates that further chain elongation of *n*-butyrate to *n*-caproate was catalyzed by strain BL-6 while strain BL-4 might convert *n*-butyrate to *iso*-butyrate.

### 3.3. Genomic Heterogeneity of Strains BL-3, BL-4, and BL-6

**Figure 2:**
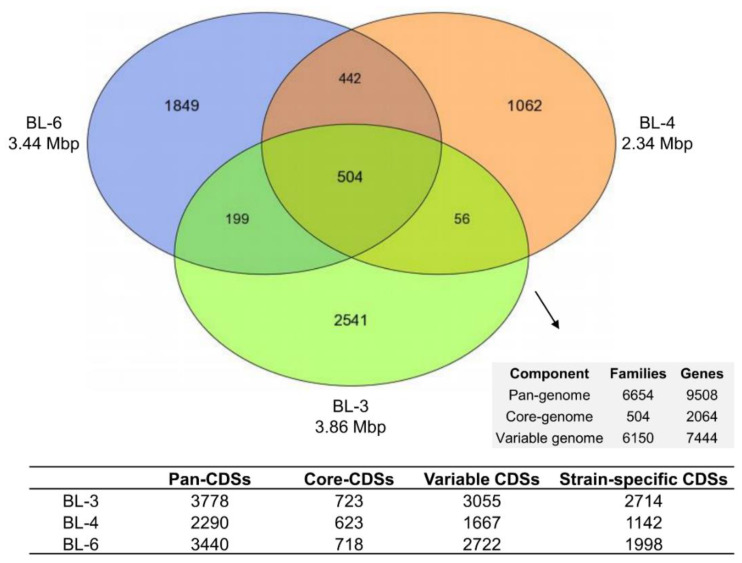
Genomic heterogeneity of strains BL-3, BL-4, and BL-6. Venn diagram showing the shared and unique gene families of the three isolates, and numbers of coding sequences (CDSs) presenting the pan-genome and core-genome as well as variable and strain-specific genes. Families refer to the MicroScope homologous gene families (MICFAM), in which the protein-coding genes share at least 80% amino acid sequence identity and 80% alignment coverage.

The genomes of all three isolates were sequenced to better understand the genetic background of their metabolism, particularly of *n*-caproate and *iso*-butyrate formation from lactate. Based on the hybrid genome assembly of short reads (Illumina) and long reads (Oxford Nanopore Technologies), we recently announced high-quality genomes of these strains with CheckM completeness of 98.6%, 97.9% and 98% and contamination of 1.0%, 0.3%, and 1.3% for BL-3, BL-4, and BL-6, respectively [19]. Single circular contigs were assembled for strains BL-4 and BL-6 while the genome assembly of BL-3 resulted in seven contigs. The genome sizes are depicted in Figure 2 and detailed in Table 1. According to the taxonomic classification of GTDB, BL-3 was assigned to the genus *Clostridium*_B (*Clostridiaceae*), whereas BL-4 and BL-6 were assigned to the genera UBA4871 and *Clostridium*_E, respectively, both belonging to the *Acutalibacteraceae* (*Ruminococcaceae* according to the NCBI taxonomy). The number of predicted gene CDSs ranges from around 2300 to almost 3900 in the three genomes (Table 1). For all three genomes, most of the CDSs could be classified in COG functional categories (76% for BL-3, 75% for BL-4, and 73% for BL-6; see details in Table S1) and EGGNOG categories (86% for BL-3, 85% for BL-4 and 83% for BL-6; see details in Table S2). Comparative genome analysis revealed a total of 6654 homologous gene families with 9508 genes identified in all three genomes and indicates a relatively small core-genome size of 504 homologous gene families (Figure 2). As for the 2064 genes conserved in the core-genome, proportions of 27.2%, 20.9%, and 19.1% can be considered core CDSs of strains BL-3, BL-4, and BL-6, respectively. The core CDSs include all necessary genes involved in bioprocesses of lactate oxidation to acetyl-CoA, reverse β-oxidation, hydrogen formation, and energy conservation (see Table 2 and details in Supplemental file 2). According to the pairwise comparison of the three genomes, a few synteny groups on nucleotide level are shared (Figure S2), which indicates the low conservation of genome organization and underlines the genomic heterogeneity of the three isolates.

### 3.4. Genomic Diversity of the Reported Chain-Elongating Bacterial Strains

In addition to our newly isolated strains, we included eleven strains that have been experimentally validated of microbial chain elongation (Table 1). Two metagenome-assembled genomes (MAGs; *Candidatus* Pseudoramibacter fermentans and *Candidatus* Weimeria bifida) were also included in the comparative genome analysis because their chain elongation traits were evident from metatranscriptome analyses [46]. These 14 obligate anaerobes isolated from various environments all belong to the phylum *Firmicutes*, class *Clostridia*, and its closest phylogenetic neighbor—*Negativicutes* (here including species *Megasphaera elsdenii* and *Megasphaera hexanoica*). The genome sizes of the strains range from 2.1 Mbp to 4.7 Mbp, and the GC content varies from 32% to 55% (Table 1).

**Figure 3:**
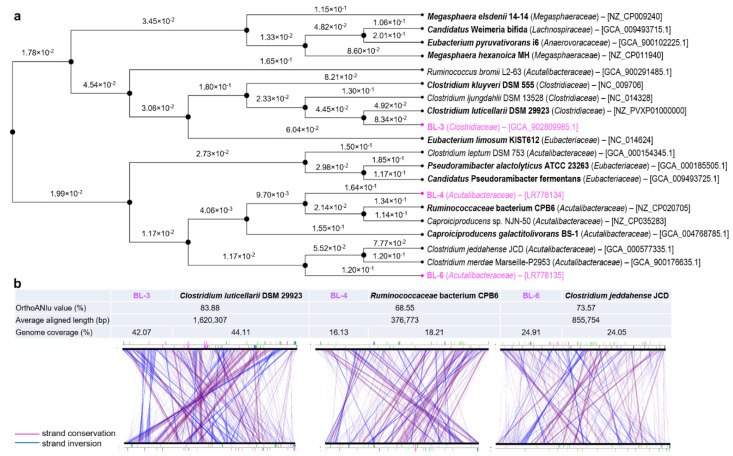
Phylogenomic analysis of the three isolates. (**a**) Neighbor-joining tree showing the genome similarity between 14 chain-elongating bacterial strains. The newly isolated strains are highlighted in pink and all experimentally validated chain-elongating strains are indicated in bold. Additional related species based on 16S rRNA phylogenetic analysis were included (see the phylogenetic tree in Figure S3). GTDB taxonomic assignments at the family level are shown in parentheses. The NCBI/ENA accession numbers of the genomes are shown in brackets. Distances indicated at the branches correlate to the average nucleotide identity (ANI) according to: D ≈ 1-ANI. (**b**) USEARCH OrthoANI comparison for strains BL-3, BL-4, and BL-6 to related genomes. The line plots give an overview of the conservation of synteny groups on nucleotide level. Strand conservations are depicted in purple and strand inversions in blue. The synton size was selected with higher than three genes for the analysis.

We constructed a phylogenomic tree to understand the evolutionary relationships between our isolates and other chain-elongating species (Figure 3a). The two main branches delineate that strain BL-3 is evolutionary distant from BL-4 and BL-6, as the latter were placed in the other main cluster. BL-3 belongs to a *Clostridiaceae* cluster and is closely related to two chain-elongating species of the genus *Clostridium*: *C. kluyveri* and *C. luticellarii*. The latter has the highest OrthoANIu (average nucleotide identity by orthology with USEARCH) value of 83.88% to BL-3 (Figure 3b), which suggests BL-3 being a new species. The closest chain-elongating relatives of BL-4 and BL-6 are *Ruminococcaceae* bacterium CPB6 and *Caproiciproducens galactitolivorans* BS-1, both affiliated to the family *Acutalibacteraceae* (according to GTDB taxonomy). BL-6 formed a separate cluster together with *Clostridium jeddahense* and *Clostridium merdae*, for which chain elongation functions have not been described. However, BL-4 and BL-6 have relatively low OrthoANIu values (≤75%) and low genome coverages (≤25%, referring to the aligned genome fraction) with their closest relatives (Figure 3b), which indicates their distant phylogenomic relationship. For all three isolates, the synteny groups on nucleotide level delineate a low conservation of genome organization when aligned to the closest relative.

**Figure 4:**
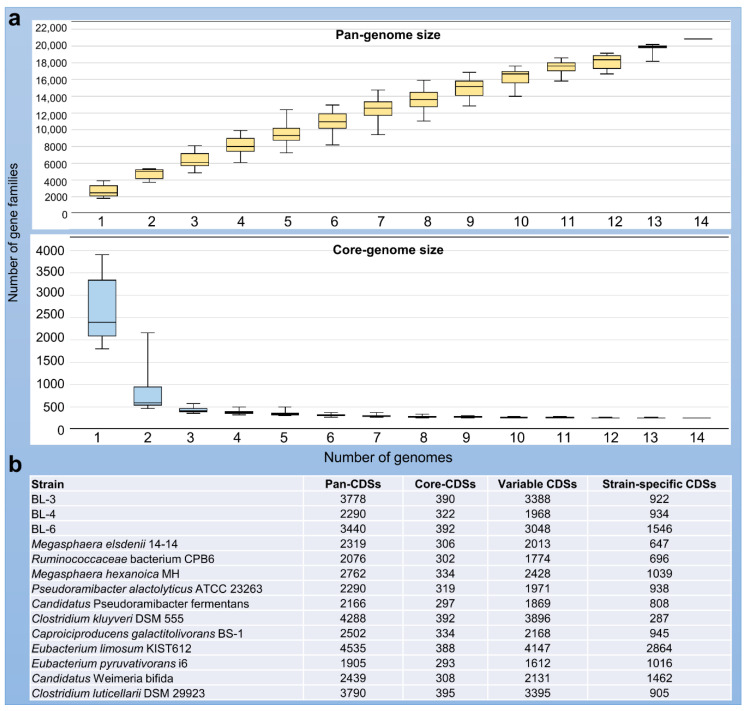
Pan-genome analysis of the 14 chain-elongating bacterial strains. (**a**) Pan-genome and core-genome sizes and their changes for the increasing genome set. Families refer to the MicroScope homologous gene families (MICFAM), in which the protein-coding genes share at 80% of amino acid sequence identity and 80% of alignment coverage. (**b**) Summary of gene counts for each strain. CDS: gene coding sequence.

The number of predicted CDSs in the chain-elongating bacteria ranges from less than 2000 to more than 4600 (Table 1), which suggests substantial heterogeneity of their genomes. The pan-genome analysis of the genomes of all 14 strains revealed a total of 20,790 homologous gene families with 40,582 genes identified (Figure 4a). The core-genome presented in all 14 strains consists of only 237 conserved homologous gene families corresponding to 4775 core CDSs, which were distributed in a range of 9% to 15% for each strain (Figure 4b). Interestingly, the number of pan-CDSs positively correlated with the genome size, whereas the number of strain-specific CDSs did not correlate with the genome size. For example, *C. kluyveri* DSM 555 holds the second largest genome (4.02 Mbp) with a number of 4288 pan-CDSs, but it has the lowest number of strain-specific CDS (287 CDSs). The above-mentioned patterns also apply to the comparison of the three isolates as shown in Figure 4b.

Functional distribution of homologous gene families in the core-genome shows that the majority encode components of well-conserved housekeeping genes for the basic metabolism of bacteria, including DNA and RNA metabolism, protein processing, folding and secretion, cellular processes, as well as intermediary and energy metabolism (details in Supplemental file 3) [60]. The chain elongation-specific core-genome also comprises genes involved in reverse β-oxidation, hydrogen formation and energy conservation (Table 2 and details in Supplemental file 4). These genes are highly conserved in all 14 strains and can be considered hallmarks of chain-elongating bacteria.

### 3.5. Genetic Basis of Lactate Conversion to n-Caproate and iso-Butyrate

To elucidate the genetic background of lactate metabolism and fermentation pathways leading to the formation of *n*-caproate, *n*-butyrate, and *iso*-butyrate, we manually curated the functional annotation of genes involved in the following bioprocesses: acetyl-CoA formation from lactate and ethanol, reverse β-oxidation cycle, energy conservation and hydrogen formation. Besides our newly isolated strains, we also included the other eleven chain elongators in this analysis. Especially for those strains reported to use lactate as electron donor, corresponding genes of lactate oxidation were also considered in the manual curation.

#### 3.5.1. Lactate Oxidation to Acetyl-CoA

**Figure 5:**
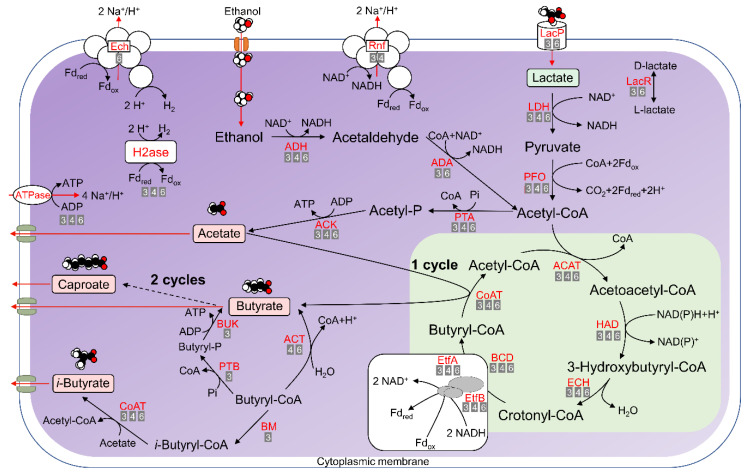
Metabolic pathways involved in lactate-based or ethanol-based chain elongation and production of acetate, *n*-butyrate, *iso*-butyrate, and *n*-caproate as predicted from the genome annotation of strains BL-3, BL-4, and BL-6. Enzyme abbreviations (see Table 2 for full names) are provided in red letters next to the pathways (solid lines). The numbers below the enzyme names indicate the strains that were predicted to harbor the corresponding CDSs, i.e., “3” refers to strain BL-3, “4” refers to strain BL-4 and “6” refers to strain BL-6. The dashed line represents multi-enzyme reactions between the two indicated molecules, and “cycle” refers to the reverse β-oxidation. The conversion of the terminal acyl-CoA to the corresponding fatty acid can be catalyzed by CoAT or alternatively by ACT as shown at the example of butyrate. A third way of butyrate formation from butyryl-CoA proceeds via phosphate butyryltransferase (PTB) and butyrate kinase (BUK). The predicted pathway of *iso*-butyrate formation via isomerization of *n*-butyryl-CoA by butyryl-CoA:isobutyryl-CoA mutase (BM) is shown; an alternative hypothetical pathway for *iso*-butyrate formation from lactate is depicted in Figure S4 (Supplemental file 1).

**Figure 6:**
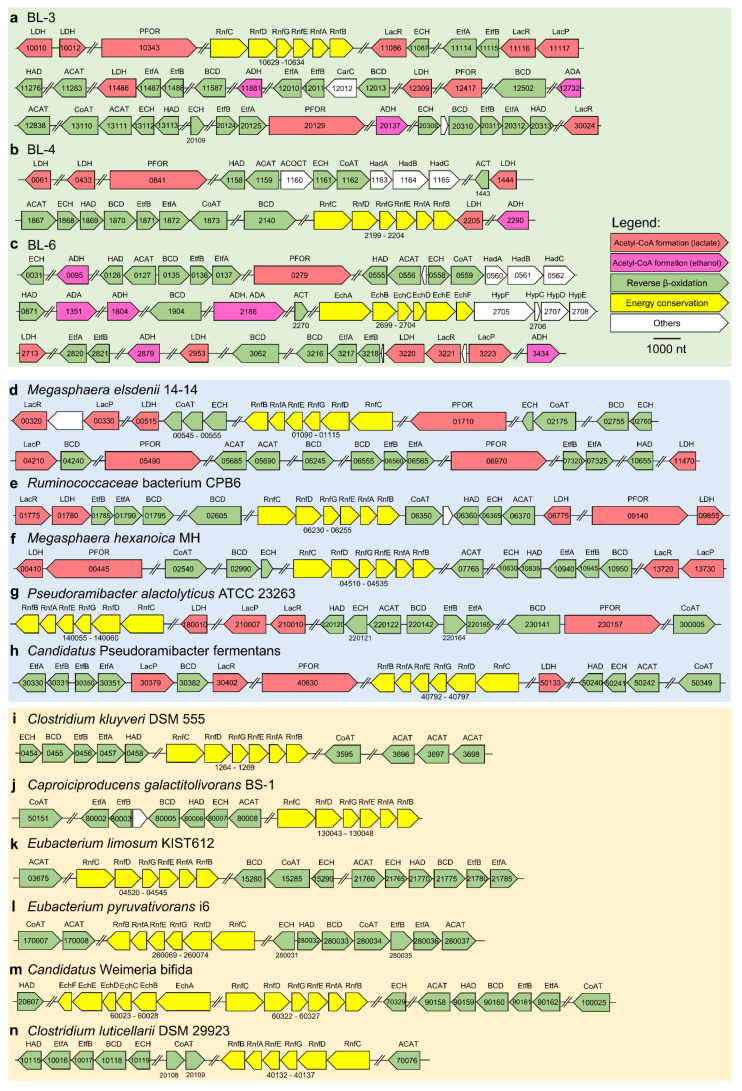
Arrangement of predicted CDSs in genomes of strains BL-3 (**a**), BL-4 (**b**), BL-6 (**c**), other bacterial strains reported of chain elongation with lactate (**d**–**h**), and with other reduced substrates (**i**–**n**). Numbers in or below the arrows denote the corresponding CDS labels. Abbreviations above the arrow refer to the enzyme names (see Table 2 for full names). Scale bar: 1000 nucleotides (nt).

Lactate can serve as a carbon and energy source for chain-elongating bacteria. As shown in Figure 5, first lactate needs to be transported into the cell, which is facilitated by lactate permease (LacP). Genomes of BL-3 and BL-6 were predicted to harbor the corresponding CDSs, which are located in a gene cluster encoding lactate racemase (LacR) (Figure 6a,c). The gene cluster encoding LacP and LacR was also found in all other lactate-based chain elongators (Figure 6d–h). The fermentation starts with the oxidation of lactate via pyruvate to acetyl-CoA catalyzed by an NAD-dependent lactate dehydrogenase (LDH) and a pyruvate ferredoxin oxidoreductase (PFOR). All three genomes encode predicted LDH proteins that are highly similar to each other. Specifically, the BL-3 genome was predicted to have four LDH genes, one of which is located in a gene cluster (Figure 6a, CDS labels: 11486–11488) comprising also genes for the electron transfer flavoprotein (EtfAB). The BL-4 genome harbors four LDH genes with one located in the gene cluster (Figure 6b, CDS labels: 2199–2205) encoding the membrane-associated energy-converting NADH:ferredoxin oxidoreductase (RnfABCDEG). The BL-6 genome has three LDH genes with one found in a cluster (Figure 6c, CDS labels: 3216–3223) including genes for butyryl-CoA dehydrogenase (BCD), EtfAB, LacR, and LacP. A similar gene cluster (Figure 6e, CDS labels: 01775–01795) containing genes for LacR, LDH, EtfAB, and BCD was found in the genome of *Ruminococcaceae* bacterium CPB6. As for the enzyme PFOR or its synonym pyruvate synthase, all three genomes contain the corresponding genes, enabling the oxidation of pyruvate to acetyl-CoA. Acetyl-CoA then enters the reverse β-oxidation cycles. CDSs for LDH and PFOR were found in all other lactate-based chain-elongating species (Figure 6d–h).

#### 3.5.2. Ethanol Oxidation to Acetyl-CoA

The ethanol-based chain elongation pathway is well elucidated in *C. kluyveri* [14] and of particular biotechnological importance as shown in several studies [61,62,63]. Genome data of BL-3 and BL-6 suggest that these strains are capable of utilizing ethanol as additional or alternative substrate. Small, uncharged molecules like ethanol diffuse through the cytoplasmic membrane and can be oxidized via acetaldehyde to acetyl-CoA. NAD-dependent alcohol dehydrogenase (ADH) and NAD(P)-dependent acetaldehyde dehydrogenase (ADA) catalyze this conversion (Figure 5). The corresponding CDSs were found in the genomes of BL-3 and BL-6, but not in the BL-4 genome.

#### 3.5.3. *n*-Butyrate and *n*-Caproate Formation

Transformation of acetyl-CoA to butyryl-CoA includes three intermediates: acetoacetyl-CoA, 3-hydroxybutyryl-CoA and crotonyl-CoA. The involved enzymes are acetyl-CoA acetyltransferase (ACAT), NAD- and NADP-dependent 3-hydroxyacyl-CoA dehydrogenase (HAD), enoyl-CoA hydratase (ECH) and NAD-dependent butyryl-CoA dehydrogenase complex (BCD/EtfAB) (Figure 5). The formation of *n*-butyrate further requires butyryl-CoA:acetate CoA transferase (CoAT) to catalyze the reaction of butyryl-CoA and acetate to yield acetyl-CoA and the corresponding fatty acid. Transformation of butyryl-CoA to caproyl-CoA may happen with the same set of enzymes (ACAT, HAD, ECH and BCD/EtfAB) and a CoAT to remove the CoA from caproyl-CoA, resulting in the formation of *n*-caproate. We came up with the same assumption as described for the ethanol-based chain elongation mechanism of *C. kluyveri* [14]—caproyl-CoA can be a further elongated acyl-CoA when a second analogous cycle proceeds, and CoAT was reported to have a broad substrate specificity [64,65]. All three genomes contain the genes encoding ACAT, HAD, ECH, BCD, EtfAB, and CoAT (Supplemental file 4 including the summary of all related CDSs). As for BL-3, three sets of ACAT, HAD, ECH, BCD, and EtfAB genes are present in the genome, with one cluster encoding CoAT, ACAT, ECH, and HAD (Figure 6a, CDS labels: 13110–13113) as well as one cluster encoding ECH, BCD, EtfAB, and HAD (Figure 6a, CDS labels: 20308–20313); other CDSs are scattered in the genome. As for BL-4, one gene cluster encoding all six enzymes is present in the genome (Figure 6b, CDS labels: 1867–1873). Two similar clusters were found in the genomes of *Eubacterium limosum* (Figure 6k, CDS labels: 21760–21785) and *Eubacterium pyruvativorans* (Figure 6i, CDS labels: 280031–280037). Another set of HAD, ACAT, ECH, and CoAT genes clusters together with the genes for acetyl-CoA:oxalate CoA-transferase (ACOCT), and (R)-2-hydroxyisocaproyl-CoA dehydratase (HadABC) (Figure 6b, CDS labels: 1158–1165).

The genome of BL-6 harbors two sets of the ACAT, HAD, ECH, BCD, and EtfAB genes separated into several sub-clusters, with one comprising genes for HAD, ACAT, ECH, CoAT, and HadABC (Figure 6c, CDS labels: 0555–0562) and two sub-clusters of genes encoding the BCD/EtfAB complex. One set of genes encoding the BCD/EtfAB complex is located in the same cluster with genes for LDH, LacR, and LacP (Figure 6c, CDS labels: 3216–3223) as mentioned above. We found that the genes encoding BCD are located in close vicinity to the genes of EtfAB in the genomes of all three isolates (Figure 6a–c), which is commonly conserved as a key feature among all genomes of other chain-elongating bacteria (Figure 6d–n).

Besides CoAT, the acyl-CoA thioesterase (ACT) may also catalyze the formation of *n*-butyrate and *n*-caproate from the terminal acyl-CoA (Figure 5). Our data suggest that the genome of BL-3 encodes proteins annotated as thioesterase superfamily proteins. We further compared their protein sequences in all the databases used (see the results in Supplemental file 5) and concluded that these thioesterases are not involved in the terminal step of reverse β-oxidation (see CDS labels and final annotations in Supplemental file 4, sheets BL-3). Genomes of BL-4 and BL-6 both contain CDSs for an ACT (see CDS labels in Supplemental file 4, sheets BL-4 and BL-6), but presenting low identity (≤ 40%) to proteins in the databases (see details in Supplemental files 6–7). Further experiments are required to assess the functionality of these CDSs and if the predicted enzymes play a role as terminal enzymes in reverse β-oxidation. A recent study on lactate-based chain elongation in mixed cultures using guild-based metabolic models suggested that butyrate is formed by CoAT, whereas caproate and caprylate are formed by ACT [66]. As pointed out by the authors, this might depend on the organisms, and the affinities of CoAT and ACT enzymes for different chain lengths need to be assessed.

Additionally to CoAT and ACT, a third pathway potentially contributing to *n*-butyrate formation from *n*-butyryl-CoA was identified in the genome of BL-3. As illustrated in Figure 5, a phosphate butyryltransferase (PTB) forms butyryl phosphate that is further converted to butyrate by a butyrate kinase (BUK). The latter step leads to the formation of one ATP, in contrast to the CoAT and the ACT routes. While the CoAT route conserves acetyl-CoA and thus obviates the need for ATP consumption in other metabolic steps where acetate needs to be activated, one can speculate that the PTB/BUK route enables higher growth rates than the CoAT route under conditions when acetyl-CoA is not limiting. Thus, the PTB/BUK route might favor butyrate production at the cost of caproate yield, i.e., butyrate is not further elongated. In our previous study on a mixed culture growing on xylan and lactate under constant conditions [18], co-occurrence network analysis predicted a *Clostridium sensu stricto* (closely related to *C. luticellarii*) as key butyrate producer that outcompeted caproate producers as reflected by higher microbial biomass production and a drop in caproate and caprylate concentrations. The lack of BUK genes in the genomes of strains BL-4 and BL-6 is consistent with the previously reported progressive loss of BUK genes found in some clostridial lineages [67]. From the biotechnological perspective, strains BL-4 and BL-6 seem to be more beneficial than BL-3 as they yield more caproate and less acetate compared with strain BL-3. However, detailed experiments are required to characterize the kinetics of lactate conversion and product formation in the strains under different growth conditions and in pure and mixed culture settings.

#### 3.5.4. Energy Conservation and Hydrogen Formation

As shown in Figure 5, the cytoplasmic BCD/EtfAB complex catalyzes the transformation of crotonyl-CoA (hexenoyl-CoA) to butyryl-CoA (caproyl-CoA) and simultaneously transfers electrons from NADH to ferredoxin, a mechanism that has been described as flavin-based electron bifurcation [68]. ATP can be produced by the ATP synthase using the ion motive force that is generated by a membrane-associated, proton-translocating ferredoxin:NAD^+^ oxidoreductase (Rnf complex) in the oxidation of ferredoxin [69]. The genomes of BL-3 and BL-4 contain the operon arranged as *rnfCDGEAB* encoding the six subunits of the Rnf complex as shown in Figure 6a,b. This gene organization (shown as *rnfBAEGDC* in the other DNA strand) was also found in other genomes of chain-elongating bacteria (Figure 6d–n). For BL-6, we could only find four genes for subunits of the Rnf complex during the functional annotation (see CDS labels in the Supplemental file 4, sheet BL-6), but it contains the CDSs encoding the analogous membrane-associated energy-converting hydrogenase (Ech complex), which was proposed to generate hydrogen for maintaining the cytoplasmic redox balance caused by the oxidation of ferredoxin [70,71]. The Ech complex uses reduced ferredoxin as electron donor and reduces protons, not NAD^+^ like Rnf. As shown in Figure 6c, CDS labels 2699–2708, a cluster encoding six subunits of the Ech complex and CDSs for the hydrogenase maturation were found. The Ech complex was also identified in the MAG of *Candidatus* Weimeria bifida (Figure 6m). Additional hydrogenases include hydrogen:ferredoxin oxidoreductase (H2ase), which was found in the genomes of all three isolates, and the bifurcating [Fe-Fe]-hydrogenase (HydABC) using electrons from NADH and reduced ferredoxin, of which no homologous genes were detected (see CDS labels in Supplemental file 4, sheets BL-3, BL-4 and BL-6).

Apart from the BCD/EtfAB complex, the predicted EtfAB-containing complexes for energy coupling may also include the LDH/EtfAB complex. The redox potential of the pyruvate/lactate pair (E_0’_ = −190 mV) is much higher than that of the NAD^+^/NADH pair (E_0’_ = −320 mV), which introduces a thermodynamic bottleneck of the lactate oxidation coupled to NAD^+^ reduction. Our annotation results show that strains BL-3, BL-6, and *Ruminococcaceae* bacterium CPB6 have LDH genes next to EtfAB genes (Figure 6a, CDS labels: 11486–11488; Figure 6c, CDS labels: 3217–3220; Figure 6e, CDS labels: 01780–01790). Therefore, similar like the mode of lactate metabolism in the strict anaerobic acetogen *Acetobacterium woodii*, we assume that the LDH/EtfAB complex of these species can also use flavin-based electron confurcation to solve the energetic enigma: driving electron flow from lactate to NAD^+^ at the cost of exergonic electron flow from reduced ferredoxin to NAD^+^ [69,72].

The manually curated annotation of all above-mentioned CDSs in the genomes of other lactate-based chain-elongating strains is provided in Supplemental file 8.

#### 3.5.5. *iso*-Butyrate Formation

The formation of *iso*-butyrate as a product of lactate-based chain elongation was experimentally proven in all three isolates. The genome analysis revealed hints on the assumed pathway, i.e., reversible *n*-butyrate/*iso*-butyrate isomerization [73,74]. As described by Matthies and Schink [74], the conversion of *n*-butyrate to *iso*-butyrate first requires activation to *n*-butyryl-CoA. Next, the isomerization of *n*-butyryl-CoA via *iso*-butyryl-CoA to *iso*-butyrate is catalyzed by a butyryl-CoA:isobutyryl-CoA mutase (BM) and an isobutyryl-CoA:acetate CoA transferase (CoAT) as shown in Figure 5. At first glance, none of the three genomes seems to encode a BM, but we found a BM homologue in the genome of BL-3 that might have been misannotated as methylmalonyl-CoA mutase. As reported by Cracan et al. [75], the fusion protein IcmF (isobutyryl-CoA mutase fused) composed of the small subunit of BM, a GTPase domain and the large subunit of BM has been widely misannotated as methylmalonyl-CoA mutase in other bacterial genomes. CDSs for a putative IcmF were found in the genomes of BL-3 and of the *iso*-butyrate producer *C. luticellarii* (see the CDS labels in Supplemental file 4). A CoA transferase gene located next to these CDSs may confirm the predicted function in isomerization. BMs catalyze the rearrangement of carboxyl groups as migration to the adjacent carbon atom, in which enzyme activities depend on coenzyme B_12_ [76]. One possible reason for the conversion of *n*-butyrate to *iso*-butyrate is that bacteria can maintain the pool of *iso*-butyrate for synthesizing valine during growth in amino acid-deficient medium [77]. As this isomerization step does not release any free energy, another possible explanation is that bacteria try to overcome inhibition effects of the accumulated *n*-butyrate, because the corresponding fatty acid of the unbranched form is more toxic than the branched form. As suggested for a methanol-based CE process [3,12], the formation of *iso*-butyrate may facilitate bacteria to further obtain energy from chain elongation.

**Figure 7:**
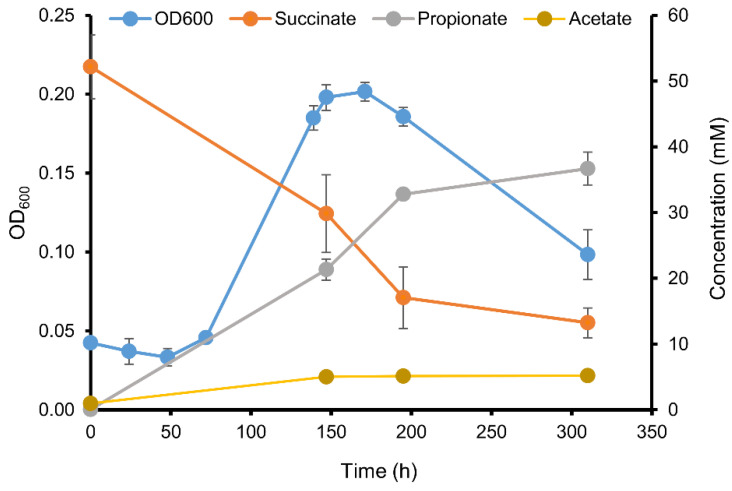
Fermentation kinetics of strain BL-4 during growth on 50 mM succinate and 0.05% yeast extract. Mean values of triplicates are shown, error bars represent the standard deviation. Some error bars are smaller than the symbol size. Small amounts (<2 mM) of formate, butyrate, *iso*-butyrate, and 1-propanol were also formed but omitted in the figure for clarity.

The genomes of BL-4 and BL-6 lack CDSs for BM, but the formation of *iso*-butyrate from lactate is also conceivable via methylmalonyl-CoA and methylmalonate-semialdehyde, representing a reverse process of anaerobic *iso*-butyrate degradation by *Desulfococcus multivorans* [78]. This hypothesis was further investigated for strain BL-4 that produces the highest *iso*-butyrate concentrations (Figure 1). At first sight, not all candidate genes predicted for this hypothetical pathway were found in the genomes of BL-4 (Figure S4,) and other reported *iso*-butyrate-producing CE species (Supplemental file 4), thus physiological experiments are needed to elucidate the mechanism of *iso*-butyrate formation in CE strains. In order to find indications of the presence of the anticipated methylmalonyl-CoA pathway, strain BL-4 was cultivated with 50 mM sodium succinate (Figure 7). The culture reached an OD_600_ of around 0.2 while concomitantly consuming 39 mM succinate and producing propionate (37 mM) and minor amounts of acetate (4.2 mM), formate (0.3 mM), *iso*-butyrate (0.2 mM), *n*-butyrate (0.1 mM), and 1-propanol (0.8 mM). Therefore, succinate was decarboxylated to propionate in an almost 1:1 stoichiometric ratio. The latter reaction, to our knowledge, is only catalyzed with the enzymes of propionic acid fermentation, i.e., via methylmalonyl-CoA as an intermediate. This indicates that BL-4 has the enzymes necessary for the conversion of organic acids to propionyl-CoA and could theoretically produce *iso*-butyrate through a reversal of the *iso*-butyrate degradation pathway in *Desulfococcus multivorans* [78]. 

**Figure 8:**
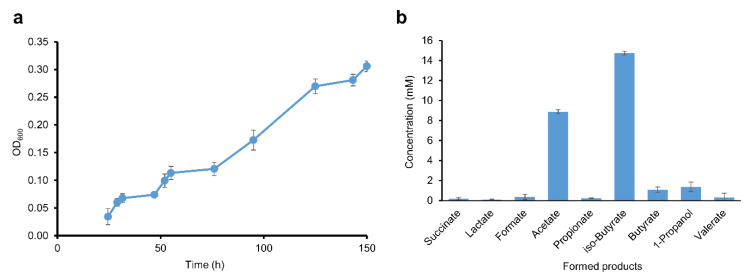
Fermentation kinetics of strain BL-4 during growth on 50 mM L-valine and 0.05% yeast extract. Mean values of triplicates are shown, error bars represent the standard deviation. Some error bars are smaller than symbol size. (**a**) optical density at 600 nm (**b**) difference of fermentation products identified and quantified by HPLC (t_end_–t_0_ values).

We hypothesize that pyruvate derived from lactate oxidation is carboxylated to oxaloacetate with concomitant decarboxylation of methylmalonyl-CoA to propionyl-CoA by a transcarboxylase. The genes for a transcarboxylase could not be identified at first sight. However, a BLAST-search of the amino acid sequence of the genes of the respective enzyme complex in *Propionibacterium freudenreichii* DSM 20271 against the genome of BL-4 revealed three potential homologs. The three major methylmalonyl-CoA carboxyltransferase subunits of *P. freudenreichii* DSM 20271 12S, 5S, and 1.3S (IMG-locus tags Ga0077868_111809, Ga0077868_111810, and Ga0077868_111807) are similar to a carboxyltransferase (CLOSBL4_v1_1895, 33% identities), an oxaloacetate decarboxylase (CLOSBL4_v1_1897, 52% identities) and a glutaconyl-CoA decarboxylase subunit gamma (CLOSBL4_v1_1896, 39% identities) respectively, and similarly arranged in one gene cluster. These genes therefore possibly constitute a methylmalonyl-CoA transcarboxylase. Yet, a gene candidate for a methylmalonyl-CoA mutase could not be identified. As a consequence of the ability to decarboxylate succinate to propionate, strain BL-4 might also be able to convert lactate to propionyl-CoA, which in turn could be carboxylated to methylmalonate-semialdehyde (MMS). MMS could then be reduced to 3-hydroxy-*iso*-butyrate (3-HIB), which then might be activated to 3-hydroxyisobutyryl-CoA (3-HIB-CoA) by a CoA-transferase. The pathway could proceed with the dehydration of 3-HIB-CoA to 3-enoyl-isobutyryl-CoA (a.k.a. methylacrylyl-CoA) and reduction of the latter to *iso*-butyryl-CoA. Finally, *iso*-butyrate could be produced either by another CoA-transferase or by phosphorylation and dephosphorylation by a phosphotransferase and an *iso*-butyrate kinase. The genes responsible for the conversion of propionyl-CoA to *iso*-butyrate could not be completely identified in the genome of strain BL-4. However, inferring from the fact that valine is degraded to acetate and *iso*-butyrate, strain BL-4 should at least have the biochemical machinery for the conversion of *iso*-butyrate to 3-HIB and methylmalonyl-CoA and vice versa (Figure 8) [79]. Otherwise, the production of acetate from valine cannot be easily explained. Acetate was always produced in media with 0.05% yeast extract (4.2 mM acetate during growth with succinate, Figure 7) and could therefore result from the degradation of other organic compounds in yeast extract. However, acetate concentrations in valine-grown cultures were twice as high (9 mM, Figure 8b). Possibly, valine could also be co-fermented in a Stickland-reaction, i.e., fermentation of pairs of amino acids such as valine and glycine, yet this would also lead to accumulation of amounts of *iso*-butyrate in a 2:1 acetate to *iso*-butyrate ratio, which was not the case (15 mM *iso*-butyrate produced, Figure 8b). It is hence unclear where the reducing equivalents derived from valine oxidation to *iso*-butyrate ended up and possibly, these reducing equivalents were used to generate the various other side products present in the valine-grown cultures (Figure 8b). Alternatively, pyruvate, and subsequently acetate, could be produced by the enzymes of the valine biosynthesis pathway acting in reverse, i.e., acetohydroxy-acid synthase (*ilvB*, CLOSBL4_v1_0646), acetolactate synthase (*ilvH*, CLOSBL4_v1_0647), and acetohydroxy-acid isomeroreductase (*ilvC*, CLOSBL4_v1_0648). Yet, it is doubtful whether the thermodynamic equilibrium allows for such a reversal of these enzyme reactions as the latter pathway usually favors valine production and at least the reaction of acetohydroxy-acid synthase is irreversible [80].

A comprehensive metabolic pathway of lactate conversion to *iso*-butyrate is not available to date for strain BL-4 and the former might be a combined variation of the known pathways of propionic acid fermentation and branched-chain amino acid degradation. It appears that *iso*-butyrate is only formed in large amounts when butyrate accumulation levels out and might also depend on the pH of the culture (Figure 1). Moreover, the amount of *iso*-butyrate formed is too high to be explained by degradation of branched-chain amino acids alone. The proposed methylmalonyl-CoA pathway could be a plausible explanation for *iso*-butyrate production from lactate, yet it remains enigmatic why strain BL-4 does not convert lactate into propionate as end-product by classical propionic acid fermentation instead of *iso*-butyrate, i.e., the question remains what are the advantages of proceeding degradation to the level of *iso*-butyrate.

## 4. Conclusions

Our results suggest three novel *Clostridia* species, represented by the strains BL-3, BL-4, and BL-6 that are able to convert lactate to *n*-caproate and *iso*-butyrate in batch cultivation, with the confirmation of their genetic background of lactate-based chain elongation and using CoA transferase as the terminal enzyme. Further research is needed to elucidate the pathways for *iso*-butyrate formation in these strains. By comparative genome analysis including further eleven experimentally validated chain-elongating bacteria, we found a substantial genetic heterogeneity but highly conserved genes related to chain elongation, hydrogen formation, and energy conservation, which can be considered hallmarks of chain-elongating bacteria. Based on the genomic features, chain-elongating species may contain two types of energy conservation systems in the re-oxidation of reduced ferredoxin—proton-translocating ferredoxin:NAD^+^ oxidoreductase (Rnf complex) and energy-converting hydrogenase (Ech complex). Besides the proposed BCD/EtfAB complex for flavin-based electron bifurcation, energy coupling may also include the LDH/EtfAB complex in the oxidation of lactate and the supply of acetyl-CoA for chain elongation. Overall, the genomic and metabolic features of the three novel chain-elongating isolates might be interesting for further research and biotechnological applications with regard to *n*-caproate and *iso*-butyrate production.

## Figures and Tables

**Table 1:** Genomic characteristics of all chain elongation strains included in this study.

Strain	GTDB Taxonomy	Isolation Source	Genome Size (bp)	GC Content (%)	No. of Predicted CDSs	Reference
BL-3	*Clostridium*_B	Anaerobic bioreactor	3,855,691	34.32	3875	[19]
BL-4	*Acutalibacteraceae* UBA4871	Anaerobic bioreactor	2,335,857	42.75	2323	[19]
BL-6	*Clostridium*_E sp002397665	Anaerobic bioreactor	3,435,529	54.63	3496	[19]
*Megasphaera elsdenii* 14-14	*Megasphaera elsdenii*	Human gut	2,504,349	52.75	2359	[47,48]
*Ruminococcaceae* bacterium CPB6	*Acutalibacteraceae* UBA4871 sp002119605	Sludge of a caproate-producing reactor	2,069,994	50.58	2116	[15,49]
*Megasphaera hexanoica* MH	*Caecibacter massiliensis*	Cow rumen	2,877,851	49.00	2799	[50]
*Pseudoramibacter alactolyticus* ATCC 23263	*Pseudoramibacter alactolyticus*	Human oral cavity	2,366,982	51.63	2327	[51,52]
*Candidatus* Pseudoramibacter fermentans ^a^	*Pseudoramibacter* sp002396065	Anaerobic bioreactor	2,288,358	50.15	2209	[46]
*Clostridium kluyveri* DSM 555	*Clostridium*_B *kluyveri*	Canal mud	4,023,800	32.02	4371	[14]
*Caproiciproducens galactitolivorans* BS-1	*Acutalibacteraceae* MS4	Anaerobic digester sludge	2,578,839	48.10	2539	[53,54]
*Eubacterium limosum* KIST612	*Eubacterium limosum*	Sheep rumen	4,740,532	46.86	4605	[51,55]
*Eubacterium pyruvativorans* i6	*Eubacterium*_A *pyruvativorans*	Sheep rumen	2,164,212	54.84	1954	[56,57]
*Candidatus* Weimeria bifida ^a^	*Lachnospiraceae* UBA2727	Anaerobic bioreactor	2,395,883	45.93	2477	[46]
*Clostridium luticellarii* DSM 29923	*Clostridium*_B *luticellarii*	Mud cellar	3,771,178	34.97	3874	[58,59]

^a^ metagenome-assembled genome (MAG).

**Table 2:** List of enzymes considered for the manual functional annotation.

Predicted Function	No.	Enzyme Abbreviation	EC Number	Enzyme
Acetyl-CoA formation	1	LacR	5.1.2.1	Lactate racemase
	2	LacP	2.A.14	Lactate permease
	3	LDH	1.1.1.27	Lactate dehydrogenase
	4	PFOR	1.2.7.1	Pyruvate ferredoxin oxidoreductase
	5	ADH	1.1.1.1	Alcohol dehydrogenase
	6	ADA	1.2.1.10	Acetaldehyde dehydrogenase
Reverse β-oxidation	7	ACAT	2.3.1.9, 2.3.1.16	Acetyl-CoA acetyltransferase
	8	HAD	1.1.1.157, 1.1.1.35	3-Hydroxyacyl-CoA dehydrogenase
	9	ECH	4.2.1.150, 4.2.1.55	Enoyl-CoA hydratase
	10	BCD	1.3.8.1	Butyryl-CoA dehydrogenase
	11	EtfAB		Electron transfer flavoprotein A,B
	12	CoAT	2.8.3.-	Butyryl-CoA:acetate CoA-transferase
	13	ACT	3.1.2.20	Acyl-CoA thioesterase
Energy conservation	14	RnfABCDEG	7.1.1.1	Energy-converting NADH:ferredoxin oxidoreductase
	15	EchABCDEF		Energy-converting hydrogenase
H_2_ formation	16	H2ase	1.12.7.2	Hydrogen:ferredoxin oxidoreductase
Butyrate formation	17	PTB	2.3.1.19	Phosphate butyryltransferase
	18	BUK	2.7.2.7	Butyrate kinase
Others	19	BM	5.4.99.13	Butyryl-CoA:isobutyryl-CoA mutase
	20	ACOCT	2.8.3.19	Acetyl-CoA:oxalate CoA-transferase
	21	HadABC	4.2.1.157	(R)-2-hydroxyisocaproyl-CoA dehydratase
	22	CarC	1.3.1.108	Caffeyl-CoA reductase-Etf complex subunit CarC
	23	HypCDEF		Hydrogenase maturation factor

## Supplementary Materials

The following are available online at https://www.mdpi.com/2076-2607/8/12/1970/s1, Supplemental File 1: Supplementary Methods, Table S1. COG (Clusters of Orthologous Groups) classification, Table S2. EGGNOG (Evolutionary Genealogy of Gene: Non-supervised Orthologous Groups) classification, Figure S1. Fermentation products of the enrichment culture (a single bottle of the fourth transfer) during growth on lactate, Figure S2. Pairwise comparison of the conservation of the synteny groups in the three new isolates, Figure S3. Maximum likelihood tree of the three new strains and closest relatives based on 16S rRNA gene sequences. Figure S4. Hypothetical *iso*-butyrate-producing lactate degradation pathway independent of *iso*-butyrate-CoA-mutase. Supplemental File 2: Data File S1, Results of pan-genome analysis of the three isolates. Supplemental File 3: Data File S2, Results of pan-genome analysis of all chain-elongating strains. Supplemental File 4: Data File S3, Annotation summary of all chain-elongating strains. Supplemental File 5: Data File S4, Functional annotations of strain BL-3. Supplemental File 6: Data File S5, Functional annotations of strain BL-4. Supplemental File 7: Data File S6, Functional annotations of strain BL-6. Supplemental File 8: Data File S7, Functional annotations of other lactate-based chain-elongating strains.
