# Supplementary material for: Three Novel Clostridia Isolates Produce n-Caproate and iso-Butyrate from Lactate: Comparative Genomics of Chain-Elongating Bacteria
Source: Microorganisms. 2020 Dec 11;8(12):1970. doi: 10.3390/microorganisms8121970 (PMC7764203; doi:10.3390/microorganisms8121970)
Supplement: Supplementary file 1 [file microorganisms-08-01970-s001.zip › microorganisms-1013833-supplementary-proofed/microorganisms-1013833-supplementary file 1 -proofed.pdf]

Supplemental File 1

Three novel *Clostridia* isolates produce *n*-caproate and *iso*-butyrate from lactate: comparative genomics of chain-elongating bacteria

Bin Liu [1], Denny Popp [1], Nicolai Müller [2], Heike Sträuber [1], Hauke Harms [1] and Sabine Kleinsteuber [1,*]

[1] Department of Environmental Microbiology, Helmholtz Centre for Environmental Research – UFZ, 04318 Leipzig, Germany; liu.bin@ufz.de (B.L.); denny.popp@ufz.de (D.P.); heike.straeuber@ufz.de (H.S.); hauke.harms@ufz.de (H.H.); sabine.kleinsteuber@ufz.de (S.K.)

[2] Department of Biology, University of Konstanz, 78457 Konstanz, Germany; nicolai.mueller@uni-konstanz.de (N.M.)

* Correspondence: sabine.kleinsteuber@ufz.de;

Supplementary methods: Sanger sequencing of 16S rRNA genes

Genomic DNA was extracted from fresh cell pellets of the isolates and purified using the NucleoSpin Microbial DNA kit (Macherey-Nagel, Germany). The concentration and quality of DNA were determined by NanoDrop™ UV-Vis spectrophotometer (NanoDrop™ ONE, Thermo Scientific, Waltham, USA) and by agarose gel electrophoresis. Amplification of bacterial 16S rRNA genes by PCR using MyTaq™ Mix (Bioline, Germany) and sequencing were carried out as described previously [81], with few modifications. For almost complete sequencing of 16S rRNA genes, sequencing primers 27f, 357f, 519r, 530f, 927r, 1104r, 1114f and 1492r were used [82]. Amplicons were purified using the SureClean Kit (Bioline, Germany) and quantified using the NanoDrop. The DNA sequence analysis software Sequencher® v5.4.6 (Gene Codes Corporation, Ann Arbor, MI USA) was used for trimming and aligning the forward and reverse sequences and assembling contigs. The sequences were compared against the National Center for Biotechnology Information (NCBI) rRNA/ITS databases (16S ribosomal RNA sequences (Bacteria and Archaea)) using the nucleotide BLAST (Basic Local Alignment Search Tool) web interface [34].

81. Sträuber, H.; Bühligen, F.; Kleinsteuber, S.; Dittrich-Zechendorf, M. Carboxylic acid production from ensiled crops in anaerobic solid-state fermentation - trace elements as pH controlling agents support microbial chain elongation with lactic acid. *Eng. Life Sci.* **2018**, 0, *447–458*, doi:10.1002/elsc.201700186.

82. Lane, D.J. 16S/23S rRNA Sequencing. In *Nucleic Acid Techniques in Bacterial Systematics*; Stackbrandt, E., Goodfellow, M., Eds.; John Wiley and Sons: Chichester, UK, 1991; pp. 177–203

Table S1. COG (Clusters of Orthologous Groups) classification

Process	Class ID	Description	BL-3		BL-4		BL-6	
			CDS	%	CDS	%	CDS	%
Cellular processes and signaling	D	Cell cycle control, cell division, chromosome partitioning	65	1.68	32	1.38	44	1.26
Cellular processes and signaling	M	Cell wall/membrane/envelope biogenesis	171	4.41	116	4.99	129	3.69
Cellular processes and signaling	N	Cell motility	71	1.83	9	0.39	68	1.95
Cellular processes and signaling	O	Posttranslational modification, protein turnover, chaperones	86	2.22	47	2.02	74	2.12
Cellular processes and signaling	T	Signal transduction mechanisms	200	5.16	82	3.53	146	4.18
Cellular processes and signaling	U	Intracellular trafficking, secretion, and vesicular transport	66	1.70	30	1.29	59	1.69
Cellular processes and signaling	V	Defense mechanisms	72	1.86	61	2.63	65	1.86
Cellular processes and signaling	W	Extracellular structures	3	0.08	6	0.26	1	0.03
Information storage and processing	B	Chromatin structure and dynamics	1	0.03	1	0.04	1	0.03
Information storage and processing	J	Translation, ribosomal structure and biogenesis	161	4.15	148	6.37	153	4.38
Information storage and processing	K	Transcription	324	8.36	196	8.44	287	8.21
Information storage and processing	L	Replication, recombination and repair	246	6.35	113	4.86	259	7.41
Metabolism	C	Energy production and conversion	271	6.99	120	5.17	168	4.81
Metabolism	E	Amino acid transport and metabolism	389	10.04	241	10.37	306	8.75
Metabolism	F	Nucleotide transport and metabolism	75	1.94	56	2.41	64	1.83
Metabolism	G	Carbohydrate transport and metabolism	177	4.57	126	5.42	263	7.52
Metabolism	H	Coenzyme transport and metabolism	131	3.38	52	2.24	109	3.12
Metabolism	I	Lipid transport and metabolism	86	2.22	61	2.63	70	2.00
Metabolism	P	Inorganic ion transport and metabolism	218	5.63	105	4.52	182	5.21
Metabolism	Q	Secondary metabolites biosynthesis, transport and catabolism	70	1.81	23	0.99	41	1.17
Poorly characterized	R	General function prediction only	483	12.46	271	11.67	349	9.98
Poorly characterized	S	Function unknown	250	6.45	154	6.63	175	5.01

Table S2. EGGNOG (Evolutionary Genealogy of Gene: Non-supervised Orthologous Groups) classification

Process	Class ID	Description	BL-3		BL-4		BL-6	
			**CDS**	**%**	**CDS**	**%**	**CDS**	**%**
Cellular processes and signaling	D	Cell cycle control, cell division, chromosome partitioning	33	0.85	25	1.08	28	0.80
Cellular processes and signaling	M	Cell wall/membrane/envelope biogenesis	168	4.34	134	5.77	126	3.60
Cellular processes and signaling	N	Cell motility	46	1.19	4	0.17	37	1.06
Cellular processes and signaling	O	Posttranslational modification, protein turnover, chaperones	91	2.35	48	2.07	65	1.86
Cellular processes and signaling	T	Signal transduction mechanisms	143	3.69	67	2.88	105	3.00
Cellular processes and signaling	U	Intracellular trafficking, secretion, and vesicular transport	38	0.98	23	0.99	37	1.06
Cellular processes and signaling	V	Defense mechanisms	72	1.86	56	2.41	67	1.92
Cellular processes and signaling	W	Extracellular structures	1	0.03	0	0	0	0
Information storage and processing	B	Chromatin structure and dynamics	1	0.03	1	0.04	1	0.03
Information storage and processing	J	Translation, ribosomal structure and biogenesis	157	4.05	147	6.33	146	4.18
Information storage and processing	K	Transcription	267	6.89	159	6.84	230	6.58
Information storage and processing	L	Replication, recombination and repair	221	5.70	111	4.78	261	7.47
Metabolism	C	Energy production and conversion	267	6.89	112	4.82	150	4.29
Metabolism	E	Amino acid transport and metabolism	315	8.13	199	8.57	261	7.47
Metabolism	F	Nucleotide transport and metabolism	76	1.96	59	2.54	65	1.86
Metabolism	G	Carbohydrate transport and metabolism	129	3.33	92	3.96	204	5.84
Metabolism	H	Coenzyme transport and metabolism	109	2.81	38	1.64	93	2.66
Metabolism	I	Lipid transport and metabolism	75	1.94	56	2.41	62	1.77
Metabolism	P	Inorganic ion transport and metabolism	158	4.08	89	3.83	185	5.29
Metabolism	Q	Secondary metabolites biosynthesis, transport and catabolism	41	1.06	14	0.60	19	0.54
Poorly characterized	S	Function unknown	979	25.26	559	24.06	782	22.37

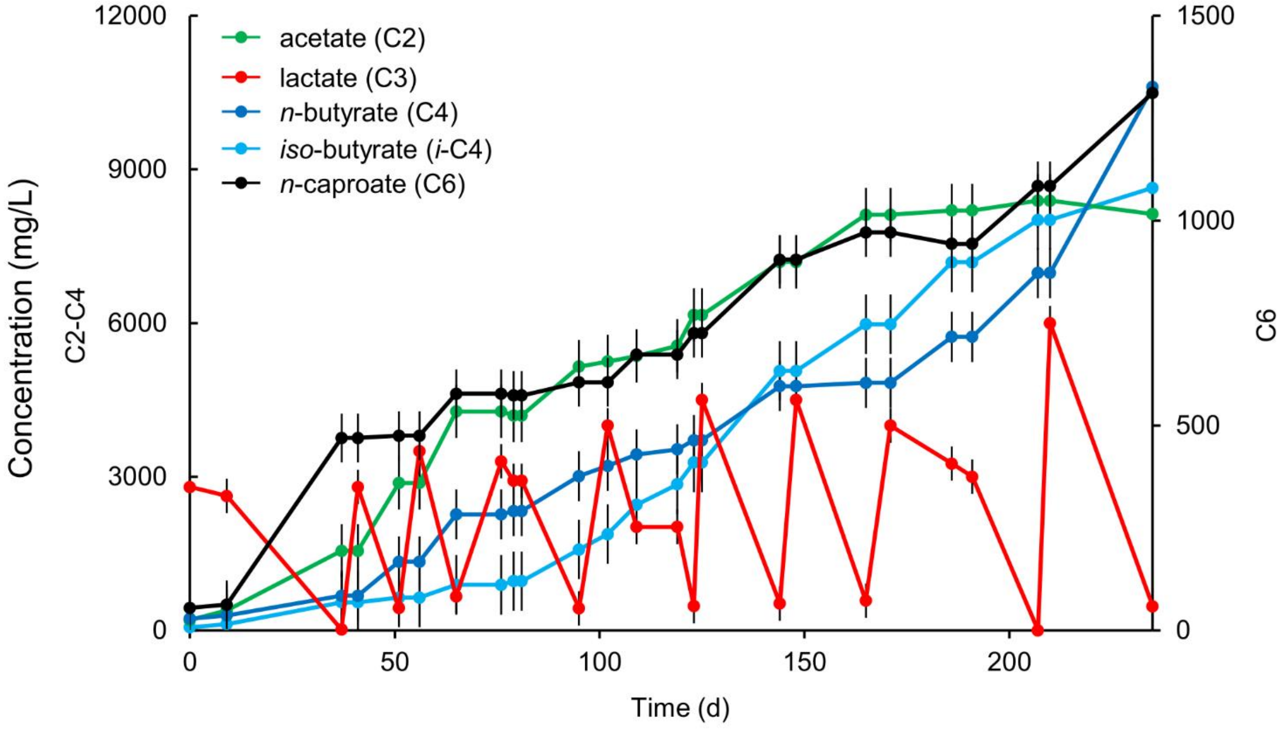

Figure S1. Fermentation products of the enrichment culture (a single bottle of the fourth transfer) during growth on lactate. Mean values of three measurements are given and error bars represent the standard deviation.

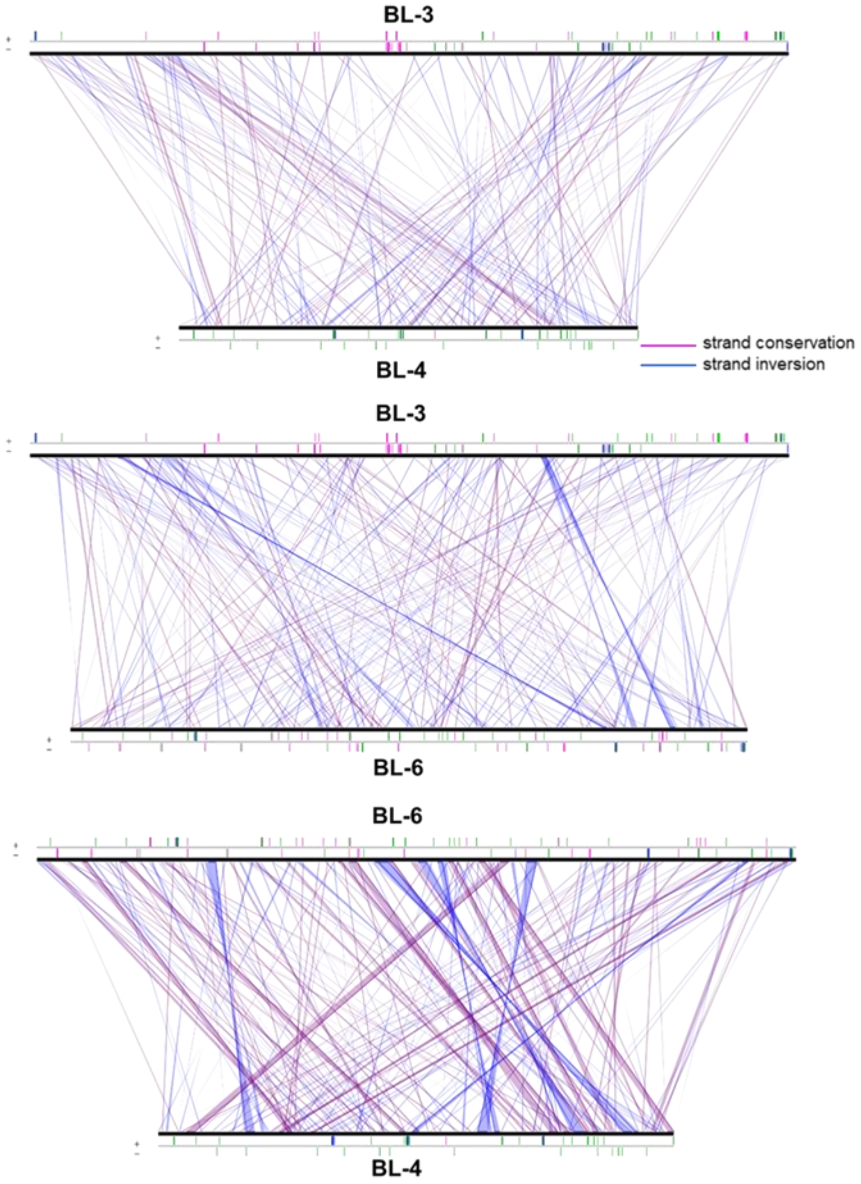

Figure S2. Pairwise comparison of the conservation of the synteny groups in the three new isolates. Strand conservations are depicted in purple and strand inversions in blue. The synton size was selected higher than three genes.

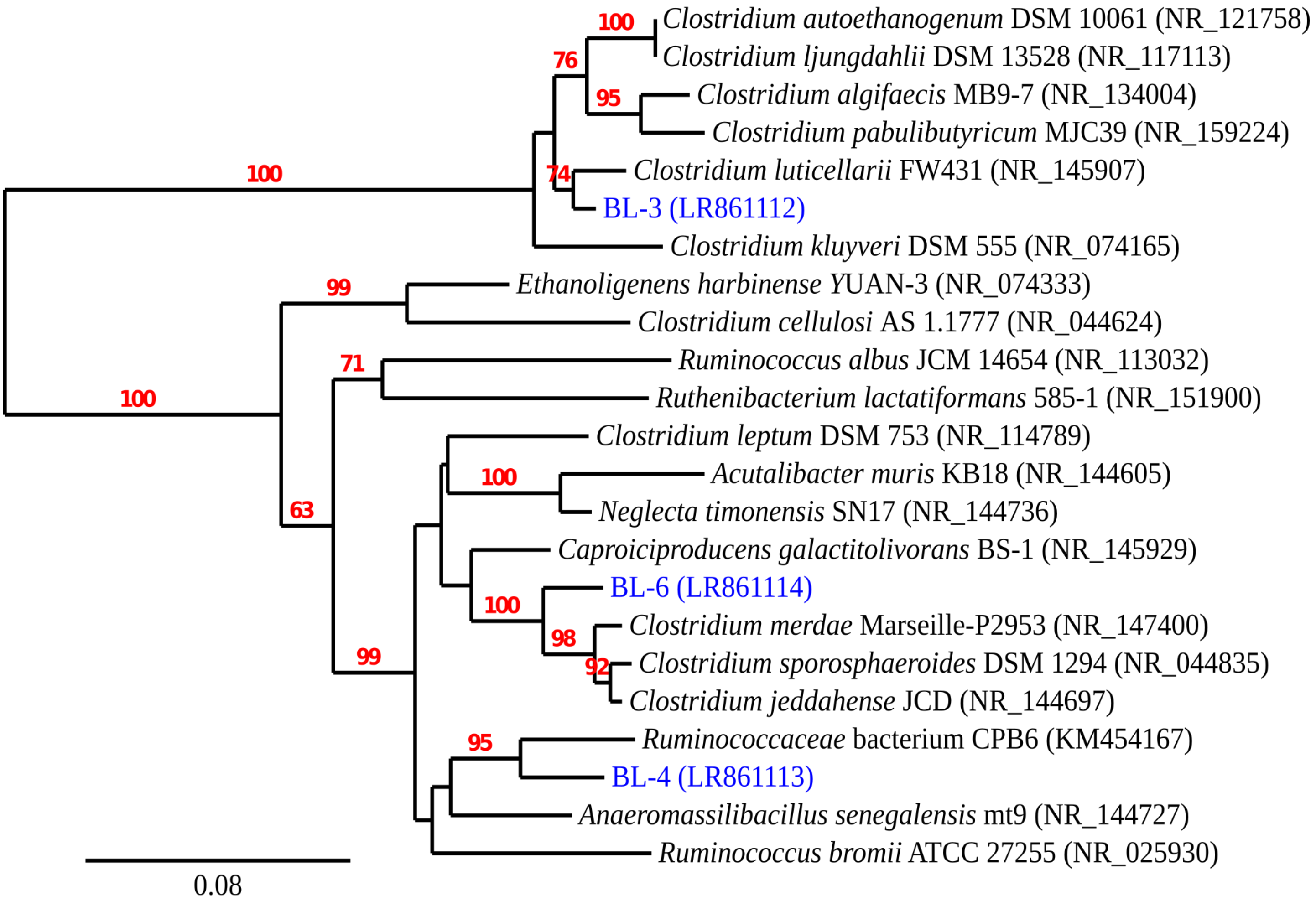

Figure S3. Maximum likelihood tree of the three new strains and closest relatives based on 16S rRNA gene sequences. Bootstrap values above 50% are shown at the node. GenBank or European Nucleotide Archive (ENA) accession numbers of 16S rRNA sequences are presented in parentheses. Scale bar = 8% nucleotide substitution.

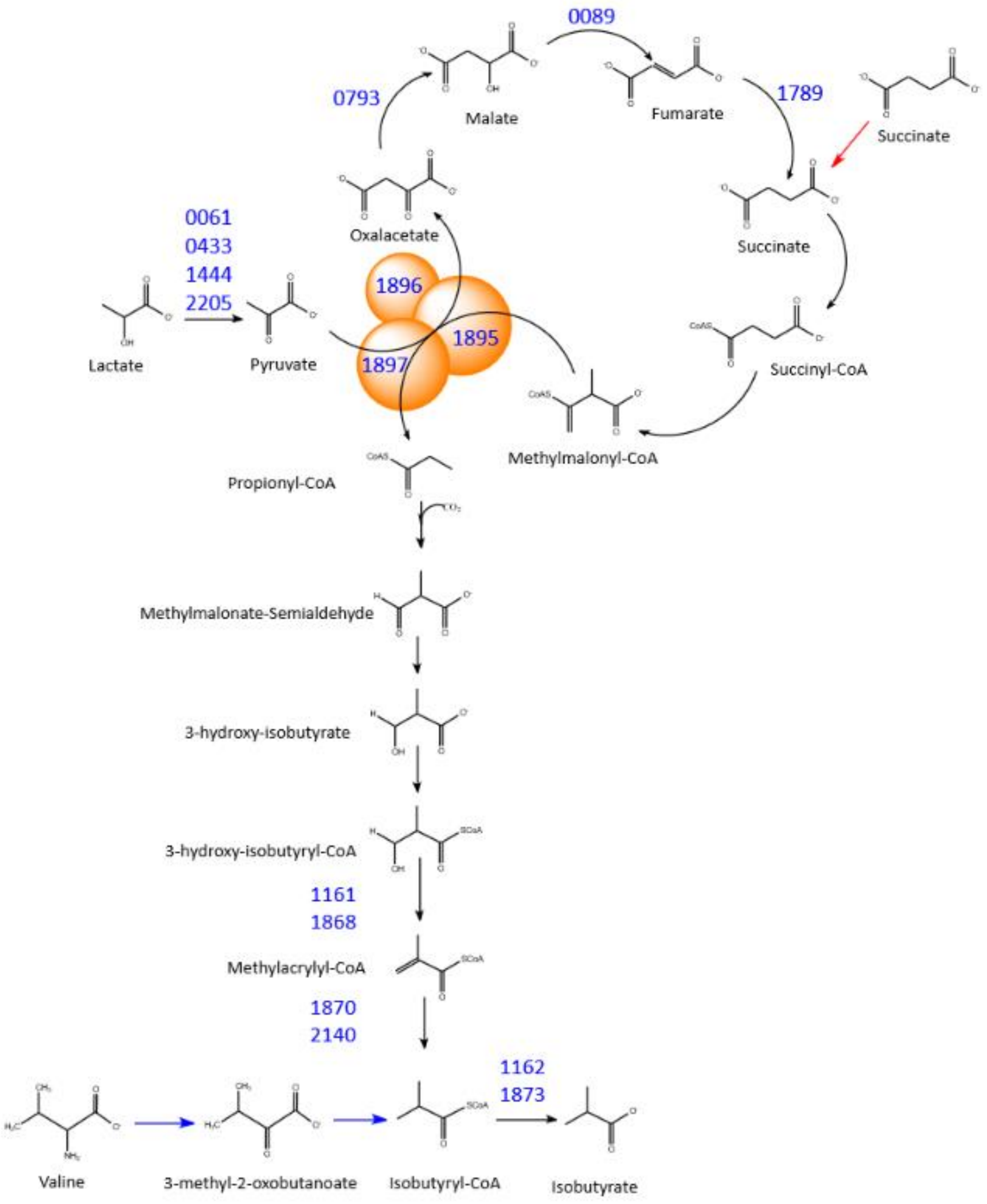

Figure S4. Hypothetical *iso*-butyrate-producing lactate degradation pathway independent of *iso*-butyrate-CoA-mutase. Numbers represent the locus tags of the predicted genes for strain BL-4. See the corresponding CDSs in Supplementary file 4.
